# Level of Thyroid-Stimulating Hormone (TSH) in Patients with Acute Schizophrenia, Unipolar Depression or Bipolar Disorder

**DOI:** 10.1007/s11064-014-1305-3

**Published:** 2014-04-11

**Authors:** Adam Wysokiński, Iwona Kłoszewska

**Affiliations:** Department of Old Age Psychiatry and Psychotic Disorders, Medical University of Lodz, Czechosłowacka 8/10, 92-216 Lodz, Poland

**Keywords:** Thyroid-stimulating hormone, Schizophrenia, Depression, Bipolar disorder

## Abstract

The aim of this study is to investigate differences in thyroid-stimulating hormone (TSH) level in patients with acute schizophrenia, unipolar depression, bipolar depression and bipolar mania. Serum level of TSH was measured in 1,685 Caucasian patients (1,064 women, 63.1 %; mean age 46.4). Mean serum TSH concentration was: schizophrenia (n = 769) 1.71 μIU/mL, unipolar depression (n = 651) 1.63 μIU/mL, bipolar disorder (n = 264) 1.86 μIU/mL, bipolar depression (n = 203) 2.00 μIU/mL, bipolar mania (n = 61) 1.38 μIU/mL (H = 11.58, *p* = 0.009). Depending on the normal range used, the overall rate of being above or below the normal range was 7.9–22.3 % for schizophrenia, 13.9–26.0 % for unipolar depression, 10.8–27.6 % for bipolar disorder, 12.2–28.5 % for bipolar depression, and 11.4–24.5 % for bipolar mania. We have also found differences in TSH levels between the age groups (≤20, >20 years and ≤40, >40 years and ≤60 years and >60 years). TSH level was negatively correlated with age (r = − 0.23, *p* < 0.001). Weak correlations with age have been found in the schizophrenia (r = − 0.21, *p* < 0.001), unipolar depression (r = − 0.23, *p* < 0.001), bipolar depression (r = − 0.25, *p* = 0.002) and bipolar disorder (r = − 0.21, *p* = 0.005) groups. Our results confirm that there may be a higher prevalence of thyroid dysfunctions in patients with mood disorders (both unipolar and bipolar) and that these two diagnostic groups differ in terms of direction and frequency of thyroid dysfunctions.

## Introduction

Thyroid-stimulating hormone (also known as thyrotropin, TSH) stimulates the thyroid gland to produce thyroxine (T4) and triiodothyronine (T3), which is a metabolism-stimulating hormone. TSH is synthesized and secreted by thyrotrope cells in the anterior pituitary gland and regulates the endocrine function of the thyroid gland. Production and secretion of TSH is stimulated by the hypothalamus, which produces thyrotropin-releasing hormone (TRH). Production of TSH is inhibited by somatostatin, which is also produced by the hypothalamus, and via a negative feedback loop by T3 and T4 [1].

Schizophrenia, bipolar disorder and unipolar depression are the most severe psychiatric disorders. They have high prevalence, chronic course, significant mental and somatic comorbidity and very high personal and societal costs (lost productivity and increased medical expenses). Also, many patients respond poorly to medications and have frequent and disrupting relapses. Finally, although several biological markers have been identified, such as cortisol [2] or brain derived neurotrophic factor (BDNF) [3], none of them is conclusive, partly because they are shared by different disorders. This results mainly from complex mechanisms underlying development of these disorders. TSH also cannot be used as a single biomarker of any mental disorder, but could serve as an additional biomarker for improving diagnostic or therapeutic procedures.

The relation between thyroid function and mental disorders has long been recognized. Thyroid disorders, including both hypothyroidism and hyperthyroidism, may be accompanied by various neuropsychiatric manifestations, ranging from depression [4] and anxiety [5] to psychosis [6]. Hypothyroidism clinical symptoms may mimic melancholic depression and dementia, while in elderly patients hyperthyroidism may mimic depression and pseudodementia [7]. Also, TSH levels are correlated with depression severity [8] and hyperthyroidism may increase the risk of developing bipolar disorders [9]. Placidi et al. [10] have found higher rates of panic disorder, simple phobia, obsessive–compulsive disorder, major depressive disorder, bipolar disorder and cyclothymia in thyroid patients than in the general population. These findings would suggest that the co-occurrence of psychiatric and thyroid diseases may be the result of common biochemical abnormalities. Association between thyroid function and mood disorders are particularly important in elderly patients. Chueire et al. [11] reported that depression was observed more frequently among individuals with subclinical (49.7 %) hypothyroidism than among individuals with overt hypothyroidism (16.8 %) (*p* < 0.001) and subclinical hypothyroidism increased the risk for a patient to present depression more than four times (OR = 4.9). In schizophrenia results of thyroid function studies are inconclusive. Rinieris et al. [12] found that antipsychotics may affect serum thyroid hormones levels. Rao et al. [13] concluded that increased dopaminergic activity affects pituitary secretory functions and may lead to reduced TSH levels. Subclinical hypothyroidism may be present in treatment-naive patients with schizophrenia and treatment with antipsychotics may increase basal TSH levels [14], while Rao et al. [15] found no difference between treated and untreated patients. Higher basal TSH levels may be associated with a poorer treatment response in schizophrenia [16], while T4 levels showed a positive correlation with the severity of illness and the degree of clinical response to neuroleptic treatment [17].

The above findings would suggest that the co-occurrence of psychiatric and thyroid diseases may be the result of common biochemical abnormalities. Therefore, we have carried out this study in order to investigate differences in TSH level in patients with schizophrenia, unipolar depression, bipolar depression and bipolar mania.

## Methods

This was a retrospective, cross-sectional, naturalistic study. Our psychiatry clinical hospital database was screened for serum thyroid-stimulating hormone (TSH) level. Only the first entry for each patient from inpatient care units were used for analysis. Usually the first blood tests are done next day after admission to our units. Thus, we have assumed that most patients that we included in the study were in acute phase of their disorder. We focused on patients with schizophrenia (all subtypes), bipolar disorder and unipolar depression. Patients of all ages were included in the study.

Results for 1,685 Caucasian patients were included in the study. Patients were grouped under diagnostic criteria as schizophrenia (F20 according to ICD-10, 295 according to DSM-IV), unipolar depression (F31 and F32 according to ICD-10, 296.2 and 296.3 according to DSM-IV), bipolar disorder (F30 and F31 according to ICD-10, 296.[0,4,6] according to DSM-IV), bipolar depression (F31.3–F31.5 according to ICD-10, 296.6 according to DSM-IV) and bipolar mania (F30 and F31.0–F31.2 according to ICD-10, 296.0 and 296.4 according to DSM-IV). In our unit diagnosis is based on the ICD-10 criteria, DSM-IV codes were given as reference.

Blood samples were drawn for all patients between 8 and 9 a.m. after 12 h overnight fast. Immediately after collecting blood samples TSH serum level was determined using automatic analyzer Dirui CS-400 (Dirui, China). For the normal range three different criteria were used: (1) the currently accepted 0.4–5.0 μIU/mL range; (2) the 0.3–3.0 μIU/mL range proposed by the American Association of Clinical Endocrinology [18]; and (3) the 0.4–2.5 μIU/mL range recommended by The National Academy of Clinical Biochemistry (NACB) [19].

Statistical procedures were performed with STATA 13.1 (StataCorp, USA). Simple descriptive statistics (means and standard deviations) were generated for continuous variables. For discrete variables number of patients and percentages are given. Normality of distribution was tested with Shapiro–Wilk test. TSH level did not follow normal distribution, even after transformation of this variable. Inter-group differences were analyzed using Kruskal–Wallis test (for TSH level) or one-way ANOVA (for age), intra-group differences were analyzed using Wilcoxon rank-sum test. The difference between proportions was analyzed with the Chi square test. Associations were tested by Spearman’s correlation coefficient. The significant level was set at *p* < 0.05.

## Results

**Fig. 1:**
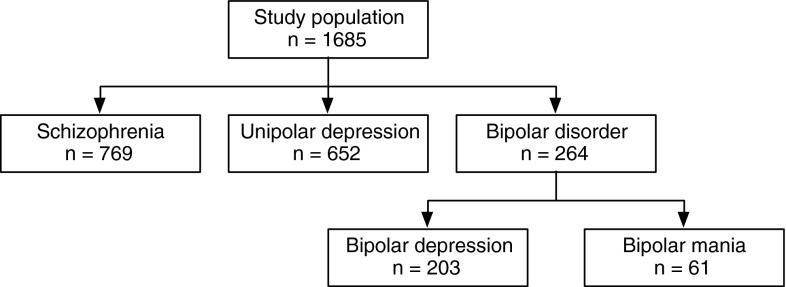
Total number of patients and stratification of patients into diagnostic groups

Ine the study group of 1,685 patients included in the analysis there were 63.1 % women (n = 1,064). The proportion of women in the schizophrenia group (n = 769) was 50.5 % (n = 388), in the unipolar depression group (n = 652) was 75.8 % (n = 494), in the bipolar disorder group (n = 264) was 68.9 % (n = 182), in the bipolar depression group (n = 203) was 71.9 % (n = 146) and in the bipolar mania (n = 61) was 59.0 % (n = 36). The difference between the groups in the proportion of women was significant (χ^2^ = 105.01, *df* = 3, *p* < 0.001), with the lowest proportion of women in the schizophrenia group. In the schizophrenia group (n = 769) there were three patients with hebephrenic subtype, 20 patients with paranoid schizophrenia and catatonic symptoms, four with residual schizophrenia and two with simple-type schizophrenia. These subgroups were to small to include them into separate analysis and therefore we decided to combine all patients with schizophrenia into one group. Figure 1 shows the total number of patients and stratification of patients into diagnostic groups.

The age (mean ± standard deviation) of the study group was 46.4 ± 19.8 years. Mean age in the subgroups was: schizophrenia 40.0 ± 16.2, unipolar depression 52.1 ± 21.7, bipolar depression 52.8 ± 18.4, bipolar mania 45.3 ± 19.8 years. One-way ANOVA showed that there were significant age differences between the groups (F = 56.72, *df* = 3, *p* < 0.001). Post hoc comparisons using the Bonferroni test showed that patients with unipolar- and bi-polar depression groups were significantly older (*p* < 0.001 for both groups). Age distribution in the study sample is shown in Table 1.

**Table 1:** Age distribution in the study sample

Diagnosis	Age category				Total [n (%)]
	<20	20–40	40–60	>60	
Schizophrenia					
Men	26	248	76	31	381 (49.5)
Women	31	172	115	70	388 (50.5)
Total	57	420	191	101	769
Unipolar depression					
Men	24	20	57	57	158 (24.2)
Women	93	34	165	202	494 (75.8)
Total	117	54	222	259	652
Bipolar disorder					
Men	11	24	21	26	82 (31.1)
Women	8	43	61	70	182 (68.9)
Total	19	67	82	96	264
Bipolar depression					
Men	5	16	12	24	57 (28.1)
Women	4	35	50	57	146 (71.9)
Total	9	51	62	81	203
Bipolar mania					
Men	6	8	9	2	25 (41.0)
Women	4	8	11	13	36 (59.0)
Total	10	16	20	15	61

We have found several significant correlations between age and TSH level. In the whole study group TSH level was negatively correlated with age (r = −0.23, *p* < 0.001), but not in the bipolar mania (*p* = 0.07) group. Weak correlations with age have been found in the schizophrenia (r = −0.21, *p* < 0.001), unipolar depression (r = −0.23, *p* < 0.001), bipolar depression (r = −0.25, *p* = 0.002) and bipolar disorder (r = −0.21, *p* = 0.005) groups.

**Fig. 2:**
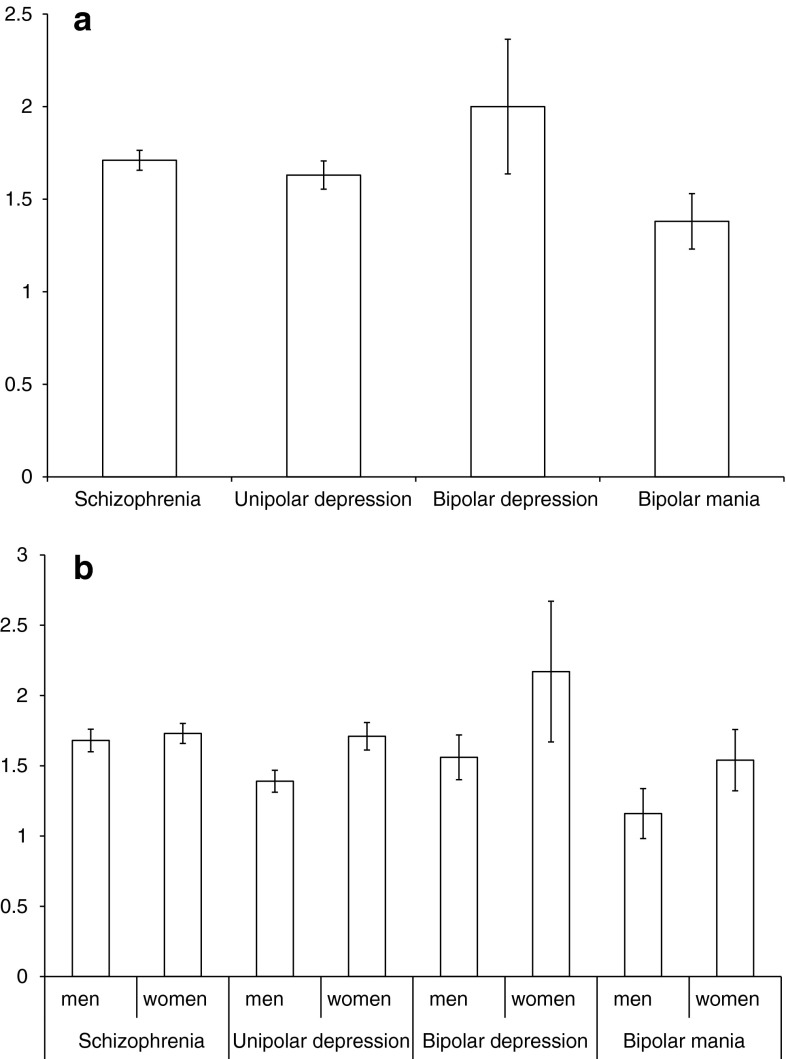
Mean TSH levels [μIU/mL] with standard error in subjects with schizophrenia, unipolar depression, bipolar depression and bipolar mania

**Fig. 3:**
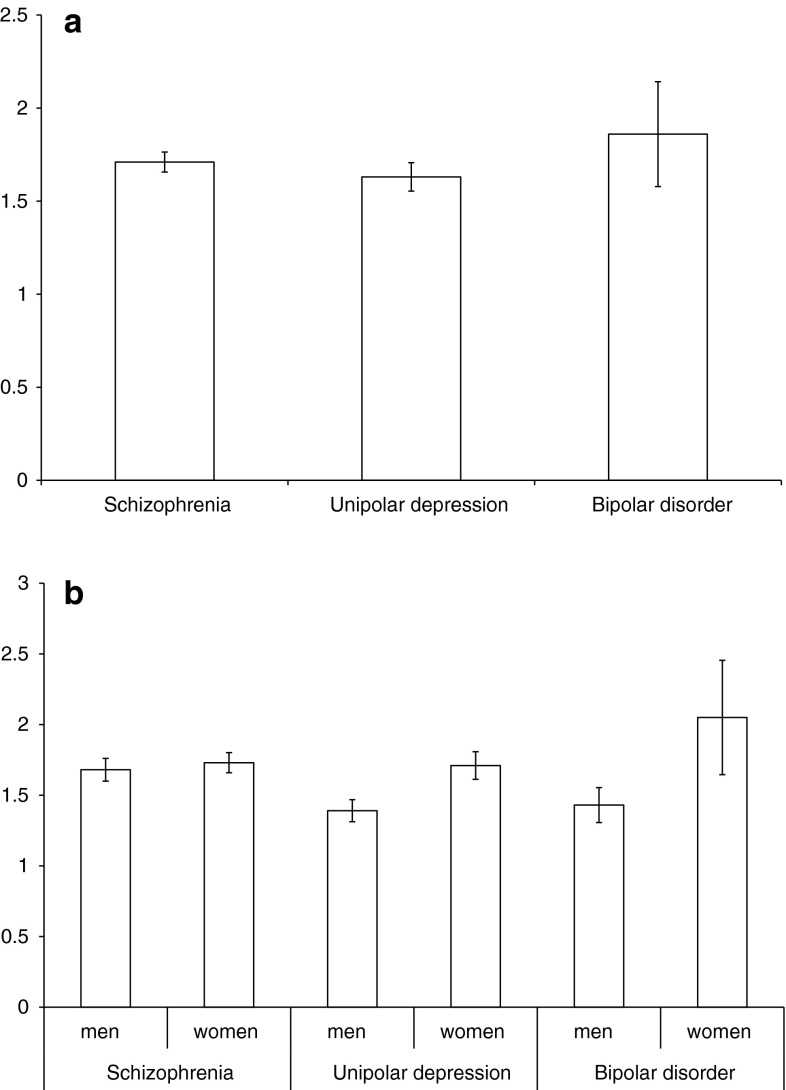
Mean TSH levels [μIU/mL] with standard error in subjects with schizophrenia, unipolar depression and bipolar disorder

For the whole study group there was no difference between men and women for TSH levels (1.58 ± 1.39 vs. 1.77 ± 2.82 μIU/mL, z = −1.13, *p* = 0.25). Mean serum TSH level in the study groups was: schizophrenia 1.71 ± 1.49 μIU/mL, unipolar depression 1.63 ± 1.95 μIU/mL, bipolar disorder group 1.86 ± 4.58 μIU/mL, bipolar depression 2.00 ± 5.18 μIU/mL, bipolar mania 1.38 ± 1.17 μIU/mL, see Fig. 2. There was a significant difference between the serum level of TSH between patients with schizophrenia, unipolar depression, bipolar depression and bipolar mania (H = 11.58, *df* = 3, *p* = 0.009). The highest level of TSH was found in the bipolar depression group (2.00 ± 5.18 μIU/mL) and the lowest in patients with bipolar mania (1.38 ± 1.17 μIU/mL). The difference was also significant for the subgroup of men (H = 9.89, *df* = 3, *p* = 0.019), but not for women (H = 4.93, *df* = 3, *p* = 0.17). When patients with bipolar mania or depression were combined into one group (bipolar disorder), there were also significant inter-group differences between patients with schizophrenia, unipolar depression and bipolar disorder (H = 8.62, *df* = 2, *p* = 0.013). The lowest level was in patients with unipolar depression (1.63 ± 1.95 μIU/mL) and the highest level was in patients with bipolar disorder (1.86 ± 4.58 μIU/mL), see Fig. 3. The difference was also significant for the subgroup of men (H = 7.49, *df* = 2, *p* = 0.023, but not for women (H = 4.17, *df* = 2, *p* = 0.12).

Table 2 shows sex distribution in diagnostic groups for normal ranges of TSH. Evaluation of the 0.4–5.0 μIU/mL normal range revealed significant differences between diagnostic groups with regard to serum TSH categories (χ^2^ = 16.29, *df* = 6, *p* = 0.012). When patients with bipolar depression and bipolar mania were combined into one group (bipolar disorder) the difference was also significant (χ^2^ = 14.78, *df* = 4, *p* = 0.005). The overall rate of being above or below the normal range was 7.9 % for patients with schizophrenia, 13.9 % for patients with unipolar depression, 10.8 % for patients with bipolar disorder, 12.2 % for patients with bipolar depression, and 11.4 % for patients with bipolar mania. The rate of patients being below the normal range was the highest in patients with unipolar depression (11.0 %), while the rate of patients being above the normal range was the highest in patients with bipolar depression (4.4 %).With the exception of bipolar mania, the rate of patients being below or above the normal range was higher in women (where it was higher only in patients with TSH level above the normal range). However, statistical analysis revealed that in no group the difference was significant. There was a significant difference between age of patients with TSH level below or above normal range (below 56.3 ± 17.6 years, normal range 45.5 ± 19.8 years, above 48.2 ± 19.8 years, F = 19.26, *df* = 2, *p* < 0.001). Post hoc analysis showed that patients with TSH level below the normal range were significantly older comparing to patients with TSH level above (*p* = 0.042) or within the normal range (*p* < 0.001).

**Table 2:** Sex distribution in diagnostic groups for normal ranges of TSH

Diagnosis	TSH category [(μIU/mL) n (%)]								
	<0.4	0.4–5.0	>5.0	<0.3	0.3–3.0	>3.0	<0.4	0.4–2.5	>2.5
Schizophrenia	44 (5.7)	706 (91.8)	19 (2.5)	24 (3.1)	659 (85.7)	86 (11.2)	44 (5.7)	597 (77.6)	128 (16.6)
Men	20 (5.2)	354 (92.9)	7 (1.8)	8 (2.1)	331 (86.9)	42 (11.0)	20 (5.2)	300 (78.7)	61 (16.0)
Women	24 (6.2)	352 (90.7)	12 (3.1)	16 (4.1)	328 (84.5)	44 (11.3)	24 (6.2)	297 (76.5)	67 (17.3)
Unipolar depression	72 (11.0)	561 (86.0)	19 (2.9)	47 (7.2)	541 (83.0)	64 (9.8)	72 (11.0)	482 (73.9)	98 (15.0)
Men	15 (9.5)	141 (89.2)	2 (1.3)	10 (6.3)	136 (86.1)	12 (7.6)	15 (9.5)	123 (77.8)	20 (12.7)
Women	57 (11.5)	420 (85.0)	17 (3.4)	37 (7.5)	405 (82.0)	52 (10.5)	57 (11.5)	359 (72.7)	78 (15.8)
Bipolar disorder	22 (8.3)	232 (87.9)	19 (2.5)	15 (5.7)	214 (81.1)	35 (13.3)	22 (8.3)	191 (72.3)	51 (19.3)
Men	6 (7.3)	74 (90.2)	7 (1.8)	3 (3.7)	72 (87.8)	7 (8.5)	6 (7.3)	64 (78.0)	12 (14.6)
Women	16 (8.8)	158 (86.8)	12 (3.1)	12 (6.6)	142 (78.0)	28 (15.4)	16 (8.8)	127 (69.8)	39 (21.4)
Bipolar depression	16 (7.8)	178 (87.7)	9 (4.4)	12 (5.9)	162 (79.8)	29 (14.3)	16 (7.8)	145 (71.4)	42 (20.7)
Men	2 (3.51)	53 (93.0)	2 (3.5)	1 (1.7)	50 (87.7)	6 (10.5)	2 (3.51)	45 (78.9)	10 (17.5)
Women	14 (9.59)	125 (85.6)	7 (4.8)	11 (7.5)	112 (76.7)	23 (15.7)	14 (9.59)	100 (68.5)	32 (21.9)
Bipolar mania	6 (9.8)	54 (88.5)	1 (1.6)	3 (4.9)	52 (85.2)	6 (9.8)	6 (9.8)	46 (75.4)	9 (14.7)
Men	4 (16.0)	21 (84.0)	0	2 (8.0)	22 (88.0)	1 (4.0)	4 (16.0)	19 (76.0)	2 (8.0)
Women	2 (5.6)	33 (91.7)	1 (2.8)	1 (2.8)	30 (83.3)	5 (13.9)	2 (5.6)	27 (75.0)	7 (19.4)

Evaluation of the 0.3–3.0 μIU/mL normal range revealed significant differences between diagnostic groups with regard to serum TSH categories (χ^2^ = 15.55, *df* = 6, *p* = 0.016). When patients with bipolar depression and bipolar mania were combined into one group (bipolar disorder) the difference was also significant (χ^2^ = 14.45, *df* = 4, *p* = 0.006). The overall rate of being above or below the normal range was 14.3 % for patients with schizophrenia, 17.0 % for patients with unipolar depression, 19.0 % for patients with bipolar disorder, 20.2 % for patients with bipolar depression, and 14.7 % for patients with bipolar mania. The rate of patients being below the normal range was the highest in patients with unipolar depression (7.2 %), while the rate of patients being above the normal range was the highest in patients with bipolar depression (14.3 %).With the exception of bipolar mania, the rate of patients being below or above the normal range was higher in women (where it was higher only in patients with TSH level above the normal range). However, statistical analysis revealed that in no group the difference was significant. There was a significant difference between age of patients with TSH level below or above normal range (below 57.9 ± 17.1 years, normal range 46.1 ± 19.8 years, above 43.7 ± 19.3 years, F = 16.50, *df* = 2, *p* < 0.001). Post hoc analysis showed that patients with TSH level below the normal range were significantly older comparing to patients with TSH level above (*p* < 0.001) or within the normal range (*p* < 0.001).

Evaluation of the 0.4–2.5 μIU/mL normal range revealed significant differences between diagnostic groups with regard to serum TSH categories (χ^2^ = 16.69, *df* = 6, *p* = 0.010). When patients with bipolar depression and bipolar mania were combined into one group (bipolar disorder) the difference was also significant (χ^2^ = 15.36, *df* = 4, *p* = 0.004). The overall rate of being above or below the normal range was 22.3 % for patients with schizophrenia, 26.0 % for patients with unipolar depression, 27.6 % for patients with bipolar disorder, 28.5 % for patients with bipolar depression, and 24.5 % for patients with bipolar mania. The rate of patients being below the normal range was the highest in patients with unipolar depression (11.0 %), while the rate of patients being above the normal range was the highest in patients with bipolar depression (20.7 %).With the exception of bipolar mania, the rate of patients being below or above the normal range was higher in women (where it was higher only in patients with TSH level above the normal range). However, statistical analysis revealed that in no group the difference was significant. There was a significant difference between age of patients with TSH level below or above normal range (below 56.3 ± 17.6 years, normal range 46.4 ± 19.7 years, above 42.0 ± 20.2 years, F = 24.58, *df* = 2, *p* < 0.001). Post hoc analysis showed that patients with TSH level below the normal range were significantly older comparing to patients with TSH level above (*p* < 0.001) or within the normal range (*p* < 0.001), while patients with TSH level above the normal range were younger than patients with TSH level within the normal range (*p* = 0.002).

We have also analyzed differences in TSH levels between the age groups. Patients were divided into four group: ≤20, >20 years and ≤40, >40 years and ≤60 years and >60 years. Age distributions of TSH level are shown in Table 3 for patients with schizophrenia, in Table 4 for patients with unipolar depression, in Table 5 for patients with bipolar disorder, in Table 6 for patients with bipolar depression and in Table 7 for patients with bipolar mania. There were significant differences between TSH level in age groups of schizophrenia patients (*p* < 0.001), in men (*p* < 0.001) but not in women (*p* = 0.077). Comparing men with women, TSH level was higher in women only in the subgroup of patients aged 40–60 (*p* = 0.008), while other differences were not significant. In patients with unipolar depression there were significant differences between TSH level in age groups (*p* < 0.001), both in men (*p* < 0.001) and in women (*p* < 0.001). Comparing men with women, TSH level was higher in women only in the subgroup of patients aged >60 (*p* = 0.002), while other differences were not significant. In patients with bipolar disorder there were significant differences between TSH level in age groups (*p* = 0.026), in women (*p* = 0.031) but not in men (*p* = 0.23). There were no differences in age groups between men and women with bipolar disorder. In patients with bipolar depression there were significant differences between TSH level in age groups (*p* = 0.014) but not in men (*p* = 0.06) and in women (*p* = 0.19). There were no differences in age groups between men and women with bipolar depression. In patients with bipolar mania there were no significant differences between TSH level in age groups (*p* = 0.64), also in men (*p* = 0.11) and in women (*p* = 0.07). Comparing men with women, TSH level was higher in women only in the subgroup of patients aged 20–40 (*p* = 0.012), while other differences were not significant (Table 7).

**Table 3:** Mean TSH level (in μIU/mL) in patients with schizophrenia

	Total	Age category				*p* ^†^
		<20	20-40	40-60	>60	
Men	1.68 ± 1.57	1.93 ± 0.90	1.75 ± 1.32	1.42 ± 2.30	1.64 ± 1.72	H = 29.62 *p* < 0.001
Women	1.73 ± 1.40	1.84 ± 1.03	1.81 ± 1.36	1.62 ± 1.56	1.63 ± 1.38	H = 6.84 *p* = 0.077
Total	1.71 ± 1.49	1.88 ± 0.96	1.77 ± 1.33	1.54 ± 1.89	1.63 ± 1.48	H = 27.56 *p* < 0.001
*p* ^‡^	z = − 0.87 *p* = 0.38	z = 0.68 *p* = 0.49	z = − 0.57 *p* = 0.56	z = − 2.66 *p* = 0.008	z = − 0.42 *p* = 0.66	

^†^Inter age-subgroups, Kruskal–Wallis test

^‡^Men versus women, Wilcoxon rank-sum test

**Table 4:** Mean TSH level (in μIU/mL) in patients with unipolar depression

	Total	Age category				*p* ^†^
		<20	20–40	40–60	>60	
Men	1.39 ± 0.98	2.20 ± 1.19	1.72 ± 1.04	1.37 ± 0.91	0.96 ± 0.63	H = 31.18 *p* < 0.001
Women	1.71 ± 2.17	1.98 ± 1.10	1.59 ± 1.22	1.86 ± 3.34	1.47 ± 1.22	H = 26.72 *p* < 0.001
Total	1.63 ± 1.95	2.03 ± 1.12	1.64 ± 1.15	1.74 ± 2.92	1.36 ± 1.14	H = 48.46 *p* < 0.001
*p* ^‡^	z = − 1.49 *p* = 0.13	z = 1.03 *p* = 0.30	z = 0.66 *p* = 0.51	z = − 0.04 *p* = 0.96	z = − 3.04 *p* = 0.002	

^†^Inter age-subgroups, Kruskal–Wallis test

^‡^Men versus women, Wilcoxon rank-sum test

**Table 5:** Mean TSH level (in μIU/mL) in patients with bipolar disorder

	Total	Age category				*p* ^†^
		<20	20-40	40-60	>60	
Men	1.43 ± 1.12	1.74 ± 1.02	1.53 ± 1.36	1.59 ± 1.28	1.09 ± 0.70	H = 4.21 *p* = 0.23
Women	2.05 ± 5.46	2.73 ± 2.28	1.94 ± 1.22	1.49 ± 1.37	2.51 ± 8.63	H = 8.87 *p* = 0.031
Total	1.86 ± 4.58	2.16 ± 1.69	1.80 ± 1.27	1.52 ± 1.34	2.13 ± 7.39	H = 9.28 *p* = 0.026
*p* ^‡^	z = − 0.89 *p* = 0.37	z = − 0.57 *p* = 0.56	z = − 1.73 *p* = 0.08	z = 0.48 *p* = 0.62	z = − 0.86 *p* = 0.38	

^†^Inter age-subgroups, Kruskal–Wallis test

^‡^Men versus women, Wilcoxon rank-sum test

**Table 6:** Mean TSH level (in μIU/mL) in patients with bipolar depression

	Total	Age category				*p* ^†^
		<20	20–40	40–60	>60	
Men	1.56 ± 1.20	1.90 ± 0.87	1.84 ± 1.47	1.84 ± 1.58	1.15 ± 0.69	H = 4.71 *p* = 0.19
Women	2.17 ± 6.05	3.54 ± 2.59	1.93 ± 1.27	1.63 ± 1.47	2.71 ± 9.54	H = 7.11 *p* = 0.06
Total	2.00 ± 5.18	2.63 ± 1.91	1.90 ± 1.32	1.67 ± 1.48	2.25 ± 8.02	H = 10.63 *p* = 0.014
*p* ^‡^	z = − 0.28 *p* = 0.77	z = − 0.98 *p* = 0.32	z = − 0.44 *p* = 0.65	z = 0.39 *p* = 0.69	z = − 0.45 *p* = 0.64	

^†^Inter age-subgroups, Kruskal–Wallis test

^‡^Men versus women, Wilcoxon rank-sum test

**Table 7:** Mean TSH level (in μIU/mL) in patients with bipolar mania

	Total	Age category				*p* ^†^
		<20	20–40	40-60	>60	
Men	1.16 ± 0.89	1.61 ± 1.19	0.91 ± 0.86	1.26 ± 0.63	0.33 ± 0.35	H = 5.94 *p* = 0.11
Women	1.54 ± 1.31	1.92 ± 1.92	2.01 ± 1.04	0.90 ± 0.42	1.66 ± 1.66	H = 6.77 *p* = 0.07
Total	1.38 ± 1.17	1.74 ± 1.43	1.46 ± 1.08	1.06 ± 0.54	1.48 ± 1.61	H = 1.66 *p* = 0.64
*p* ^‡^	z = −1.07 *p* = 0.28	z = 0.00 *p* = 1.00	z = −2.52 *p* = 0.012	z = 1.17 *p* = 0.23	z = −1.69 *p* = 0.09	

^†^Inter age-subgroups, Kruskal–Wallis test

^‡^Men versus women, Wilcoxon rank-sum test

## Discussion

The aim of this retrospective, cross-sectional, naturalistic study was to investigate differences in TSH level in acute phase of schizophrenia, unipolar depression, bipolar depression and bipolar mania. We have analyzed TSH level in age subgroups of diagnostic groups, as well as distribution of results within and outside three different normal ranges.

We have found that patients with bipolar disorder have the highest level of TSH, while the lowest level was seen in patients with unipolar depression. When patients with bipolar mania and bipolar depression were analyzed as separate groups, we have found that patients with bipolar depression had the highest level of TSH, while the lowest level was found in patients with bipolar mania. This was also true of a subgroup of men, but not for women. This difference cannot be explained only by differences in age between study groups since patients with unipolar- and bi-polar depression were significantly older comparing to other study groups.

The direction of TSH abnormalities (to hypothyroidism or hyperthyroidism) depended on the normal range used for analysis. When the widest normal range was used (0.4–5.0 μIU/mL) the abnormality was mainly in the direction of hyperthyroidism, while for other two ranges the abnormality was mainly in the direction of hypothyroidism. These observations were independent on diagnostic group and sex, with the exception of patients with bipolar mania, but this group was much smaller and thus under representative comparing to other diagnostic groups. Patients with bipolar depression had the highest rate of being above or below normal range of TSH level. With the exception of bipolar mania, in all diagnostic groups there were differences in TSH level between predefined age groups. In patients with schizophrenia and unipolar depression older patients had the lowest level of TSH. In patients with bipolar disorder and bipolar depression lowest TSH levels were in patients aged 40–60 (there were no significant differences between TSH level in age groups of patients with bipolar mania).

We have found that TSH levels correlated negatively with age. This is contradictory to the results of the population-based National Health and Nutrition Examination Survey (NHANES) [20], where positive correlation between age and TSH level was found in a large (n = 17,353) sample. This observation might be explained by the fact that our study sample has various potential risk factors of thyroid dysfunction, associated with clinical diagnosis and resulting from treatment. On the other hand, our results are consistent with results of the German study, which show that age was the only independent factor and was significantly inversely associated with TSH [21].

Meta-analysis of thyroid dysfunctions in Europe showed that the prevalence of undiagnosed hypothyroidism was 4.94 % and the prevalence of undiagnosed hyperthyroidism was 1.72 %. The prevalence of both previously diagnosed and undiagnosed hypothyroidism and hyperthyroidism was 3.05 and 0.75 %, respectively [22]. For depression, 1–4 % of patients are found to have overt hypothyroidism while subclinical hypothyroidism occurs in 4–40 % of these patients [11]. In 868 elderly psychiatric long-term care patients 10.8 % had elevated TSH, 8 % in those with a prior diagnosis of hypothyroidism, while low TSH levels were found in 0.07 % patients [23].

According to our data the prevalence of clinical and subclinical hypothyroidism in the study sample may be as high as 4.4–20.7 % (in patients with bipolar depression, for normal range 0.4–5.0 and 0.4–2.5 μIU/mL, respectively). As reported by Cole et al. patients with bipolar depression and with optimal thyroid profile experienced remission 4 months faster than the remainder of the study group. This study provides further evidence that patients with bipolar disorder are particularly sensitive to variations in thyroid function within the normal range and nearly three-quarters of patients with bipolar disorder have a thyroid profile that may be suboptimal for antidepressant response [24].

Regarding hyperthyroidism, the prevalence of clinical and subclinical hyperthyroidism in the study sample may be as high as 7.2–11.0 % (in patients with unipolar depression, for normal range 0.3–3.0 and 0.4–2.5 or 0.4–5.0 μIU/mL, respectively). The rate of low TSH in patients with bipolar disorder (TSH < 0.4 μIU/mL, 8.3 %) in this study was higher than previously reported (6.2 %) [25]. As noted by these authors, different definitions of hyperthyroidism are used in studies and direct comparisons are difficult.

Since we have no data on free T4 level, our interpretation of TSH level might be inaccurate and can be used only as rough estimations. Moreover, it should be noted that a state of “brain hypothyroidism” was reported in patients with depression [26]. This state of brain hypothyroidism occurs in systemic euthyroidism and may result from a defect of thyroid hormone receptor or impaired thyroid hormone transporter [27].

In the large NHANES study mean serum TSH was 1.50 μIU/mL and was higher in females than males [20]. Comparing to this result, in our study sample mean serum TSH levels were higher in patients with schizophrenia (1.71 μIU/mL), unipolar depression (1.63 μIU/mL), bipolar disorder (1.86 μIU/mL), bipolar depression (2.00 μIU/mL) and lower in patients with bipolar mania (1.38 μIU/mL). As above, this may also be explained by the fact that our study sample was not population-based and it reflects associations between mental disorders and thyroid dysfunctions.

In conclusion, our results confirm that there may be a higher prevalence of thyroid dysfunctions in patients with mood disorders (both unipolar and bipolar) and that these two diagnostic groups differ in terms of direction and frequency of thyroid dysfunctions. This also confirms the need to monitor TSH level regularly in patients with all types of mood disorders. When low normal TSH ranges are used (0.3-3.0 or 0.4-2.5 μIU/mL), we could detect subclinical thyroid dysfunctions in higher number of highly susceptible patients (lowering the normal TSH range from 0.4-5.0 to 0.4-2.5 caused a five to eight-fold increase in the number of patients who had TSH levels outside the normal range). This is particularly important in the light of recommendations of the American Association of Clinical Endocrinologists, which state that “The diagnosis of subclinical hypothyroidism must be considered in every patient with depression” [18].

Our study has some limitations, which results mainly from its retrospective and naturalistic design. We have no clinical data, data for subtypes of bipolar disorder type I and II and rapid cycling. Diagnostic groups were based on diagnosis at discharge from the hospital, so it might be inaccurate in some cases, where strict diagnostic procedure using clinical criteria was not done. We only have data representing current medical condition and have no information regarding past thyroid disorders and their treatment. We also have no date on detailed thyroid assessment (T3, T4, anti-thyroid autoantibodies, results of ultrasonograph examinations). Diagnostic groups of our study were not homogenous, there were less women in the schizophrenia group comparing to other groups, while patients with unipolar and bipolar depression were significantly older comparing to other groups. Also, the bipolar disorder group was smaller comparing to schizophrenia and unipolar disorder (this is particularly true for bipolar mania group). These factors may affect results of inter- and intra-group comparisons. In case of patients with bipolar disorder we cannot exclude the effect of treatment with lithium on TSH level. Lithium may cause hypothyroidism, it was also reported that anti-depressants [28] as well as haloperidol [29] may affect thyroid functions and we have no detailed data on treatment that could be included in the analysis. On the other hand, the large sample size and ability to compare three major clinical groups (schizophrenia, unipolar depression and bipolar disorder) are strengths of the study.
